# Book Review

**Published:** 1899-12

**Authors:** 


**The Animal as a Prime Mover.** By R. H. THURSTON. Pp. 297—338. Washington: Government Printing Office. 1898.—This is an essay, republished in separate form, from the Smithsonian Report for 1896. It deals with man from the point of view of a mechanic. The energy-value of the various food-stuffs is considered, then that of the various dietaries; and, subsequently, the various ways in which energy is dissipated from the body, such as muscular work. The vital machine is then compared and contrasted with other machines, and its efficacy discussed. Most of the facts brought forward are to be found in any standard work on physiology.

**On the Study of the Hand for Indications of Local and General Disease.** By EDWARD BLAKE, M.D. Pp. 53. London: Henry J. Glaisher. [1898.]—A considerable amount of interesting information is here given. The temperature, dryness, and moisture of the hand, its colour and texture, the varieties of nail development, the condition of the hand in various diseases, clubbed fingers, the varieties of perverted sensations, are all referred to in a manner that will bring home to the reader the utility of careful examination of the hands in the diagnosis of many diseases. Dr. Blake does not profess that this *brochure* touches much on the hand as a diagnostic factor in diseases of the nervous system, although he gives plates of the hand in acromegaly in the adult, in congenital cretinism, and in Mongol idiots, and he mentions the *main en griffe* of Duchenne. The shortening of the second phalanx of the little finger, with lateral displacement of the terminal phalanx, is interesting as being associated with certain forms of cretinism. Although fault may be found with the way in which this booklet is issued, it shows acute power of observation and cannot fail to be of use in clinical work.

**Practical Uranalysis and Urinary Diagnosis.** By CHARLES W. PURDY, M.D. Fourth Edition. Pp. xvi., 365. Philadelphia: The F. A. Davis Company. 1898.—The fact that is stated in the preface to this book, that three large editions of it have been exhausted in three years, and that it has been extensively adopted as a text-book in the United States, speaks for its value as a practical guide to the study of the urine.

This issue retains the essential features of the former editions, but has been thoroughly revised, and the chapters on the chemistry of the urine have been largely re-written, whilst a number of new illustrations have been added. It may be regarded as a trustworthy guide to the subject. We differ, however, from Dr. Purdy in his appreciation of the relative value of the clinical tests for albumin, and think the salicyl-sulphonic test might have been given. The book is well printed in excellent type, and the illustrations are good.

**Observations on Cardio-Vascular Repair.** By W. BEZLY THORNE, M.D. Pp. 26. London: J. & A. Churchill. 1898.—This paper, which was written for the 1898 meeting of the British Medical Association, deals with heart failure associated with arterial disease, and the therapeutic value of saline baths and exercises in such cases. Dr. Thorne adopts the view that defective metabolism of the body may cause disease of the arteries, and upon the vascular changes cardiac derangement may follow. Two skiagrams are given to show that the heart diminishes in size after resisted exercises. Although we believe, in some measure, in the therapeutic value of saline baths and exercises, the rapid diminution in the size of the heart does not appear to us to have been proved. We cannot see further evidence in these skiagrams. It appears to us that in the photograph in which the heart appears the smaller, there has been longer exposure to the X-rays.

**On So-called Spasmodic Asthma.** By ERNEST KINGSCOTE, M.B. Pp. 18. London: Henry J. Glaisher. 1899.—The author tells us that he is "engaged exclusively in the treatment of heart affections," and that he is "Physician in Ordinary to the Imperial Ottoman Embassy." We therefore conclude that the only maladies of the Sultan's official representatives in this country are cardiac—and this, to say the least, is extraordinary. Dr. Kingscote informs us that the effects of asthma "consist essentially in spasmodic contractions of the voluntary muscles of inspiration, and of the involuntary muscles which surround the bronchioles." Both these statements are misleading if not inaccurate. The next astonishing statement is, that "It is pretty generally conceded that the origin of asthma is to be found in the irritation of one or . . . many ramifications of the vagi; whether it be from . . . Meckel's ganglion . . . through the recurrent laryngeal . . . or through irritation of the . . . abdominal and pelvic viscera." From which it may be gathered that the author's knowledge of the anatomy and physiology of the vagus is not great. The author's "consideration of the amount of space taken up . . . by the enlarged heart" leads him to the conclusion that "the following may be instanced as direct effects of intermittent pressure." Of "the following" we may quote "brachial pain, due to perineuritis," "pain from perineuritis of the intercostals," "interference . . . through pressure on the thoracic duct," as not having a tittle of evidence in their favour. The main thesis of the paper is that a largely dilated heart, "say the size of a small football (not at all an unknown condition) . . . flops in the direction of gravity," and "can hammer" the vagus nerves on the bony spine and so cause asthma! It has rarely been our misfortune to read such a tissue of unwarrantable inferences from clinical phenomena. The progress of medicine is retarded by such publications.

**Golden Rules of Surgical Practice.** By E. Hurry Fenwick. Fifth Edition. Pp. 71. Bristol: John Wright & Co. [1898.]—Many of these rules are undoubtedly valuable, and some are not to be found in ordinary text-books; but every now and again we find one of very doubtful worth, or which conveys very imperfect information. For instance, all the directions we find given for dealing with hemorrhage are, "Always tie both ends of a divided artery in a wound." This is most important; but, surely, we might have a few rules about venous bleeding and secondary hemorrhage. Again, we are told never to hesitate or delay in opening an abscess in the loin, due to rupture or injury of the kidney; but why hesitate or delay in opening any abscess in the loin? Then the author lays it down as a rule that we are not to open a collection of pus anywhere near a large artery without first using a stethoscope. The meaning of this rule is obscure. Probably the idea is that by excluding a *bruit* we may exclude the possibility that we have, not an abscess, but a ruptured or traumatic aneurysm to deal with; but we should not like to exclude this possibility because no *bruit* was present. Before we gave the book to a student we should like to have some rules removed and others more fully elaborated. But probably no surgeon would be quite satisfied with such a series of rules drawn up by any other surgeon, and there is certainly much useful information in this waistcoat-pocket booklet.

**Drs. Harvey and Davidson's Syllabus of Materia Medica.** By William Martindale. Tenth Edition. Pp. xvi., 64. London: H. K. Lewis. 1898.—This edition is rendered more valuable than its predecessors by having quantities and doses expressed in metric as well as in imperial measurement, thus going one step further in advance than the *British Pharmacopœia*. This small work is designed to assist students in getting up the subject of Materia Medica for examination, and to this end it is admirably adapted, being a classified epitome of the *B.P.*

**The Treatment of Disease by Physical Methods.** By Thomas Stretch Dowse, M.D. Pp. xii., 412. Bristol: John Wright & Co. 1898.—This is a new edition of the author's work entitled *Lectures on Massage and Electricity in the Treatment of Disease*, and contains a fair account of the methods of massage and the use of electricity in the treatment of various diseases, but it lacks conciseness. Its bulk is due in large measure to mere padding; for instance, there is an account of Latham's theories of the constitution of proteids, and a long *verbatim* report of several of Charcot's cases of hysteria. The book is full of such superfluous matter. One does not need sketchy and incomplete descriptions of every disease in which massage may be useful. By reason of this the work is far inferior to such a treatise as Murrell's on the same subject. Life is too short for one to read such a watered account of what after all is only one method of treatment.

**The Erection of a Consumptive Sanatorium for the People.** By Dr. Nahm. Translated by William Calwell, M.D. Pp. 52. Belfast: Mayne & Boyd. 1898.—The interest which has recently been aroused in England in the question of the open-air treatment of consumption makes it all the more needful that English physicians should be familiar with the practices which have existed in many health-resorts in Germany since the establishment of Brehmer's Curhaus at Görbersdorf in 1859. It seems now to be a fashionable doctrine that "we can create a local climate through the arrangement of the buildings," and hence that we should endeavour to carry out a climatic treatment of consumption in all countries, at any elevation and at any temperature. Surely the climatic question in the treatment of phthisis is dwindling down to something like vanishing point.

**The Properties and Uses of Pure Glycerin.** By M.D., M.R.C.S. Pp. 49. [1898.]—Some years ago, at a time when pure glycerine was not such a common commodity as at present, Price's Patent Candle Company issued a pamphlet intended to draw attention to the many applications of this useful substance to medicine and pharmacy. This has been re-written, and the origin, properties, and uses of glycerine are well reviewed in half-a-dozen brief chapters, concluding with a bibliography embracing the period 1854—1897.

**The War with the Microbes.** By E. A. de Schweinitz. Pp. 485—496. Washington: Government Printing Office. 1898. —Reprinted from the Smithsonian Report, this will serve a good purpose, as Dr. de Schweinitz gives a clear and popular account of the germ theory of disease and of the system of serum-therapeutics. Attention is first drawn to the living micro-organisms, and then to those chemical products known as ptomains, enzymes, and toxins, and the specific differences of these bodies are clearly explained. The nature and method of action of antitoxins are discussed, and the paper concludes with an enumeration of the useful purposes to which germ activity is now applied.

**Golden Rules of Gynæcology.** By S. Jervois Aarons, M.D. Pp. 63. Bristol: John Wright & Co. [1898.]—This is not a book to be recommended. Without the safeguard of experience, it is likely to be productive of more harm than good. Works of this kind are not needed. Students should not be allowed to acquire their knowledge after the methods here set forth, and to the expert and operating gynæcologist the book is an impertinence.

**The Pocket Formulary for the Treatment of Disease in Children.** By Ludwig Freyberger, M.D. Pp. xv., 208. London: The Rebman Publishing Company, Limited. 1898.—There are included in this work many useful formulæ, but why the author has put the equivalents of the solid and not the liquid measures in the metric system does not appear to be very clear. The work is not merely a collection of prescriptions, but a sort of materia medica in brief, each drug having its properties, use, therapeutics, dose, and incompatibles clearly put. Useful hints are given how the taste of nauseous drugs may be disguised. It will be found a useful book.

**Informes Rendidos por los Inspectores Sanitarios de Cuartel y por los de los Distritos al Consejo Superior de Salubridad, [1896], 1897.** 2 vols. Mexico: Imprenta del Gobierno, en el ex-Arzobispado. 1898.—Previously to the year 1898 the city of Mexico, having a population of 350,000, was in a deplorably insanitary condition. There was no drainage, unless the open sewers in the town could be called so; the streets were polluted with every description of filth and garbage, and there was but little potable water. The natural consequence was that zymotic affections flourished unchecked, and the mortality from all causes reached the high figure of 58.6 per thousand. Of these the most formidable was typhus fever, which has been endemic in the city and neighbourhood since 1545, and in 1895 was responsible for nearly 2,000 deaths. Other diseases of the same class were proportionately fatal, notably smallpox and measles; but the former affection has not been so bad of late because vaccination has been most effectually carried out, there being no conscientious objectors in Mexico. At last this heavy mortality attracted the attention of the authorities, and they determined to grapple with the difficulty: for this purpose the city was divided into eight wards, and a medical man and a sanitary inspector were appointed to make a house-to-house visitation in each and report to the Council of Health on the state of things in every district. They recommended *inter alia* that a system of underground drainage should be carried out in all parts, that a number of workmen should be regularly employed in keeping the streets in a proper sanitary condition, and that water should be brought from a distance, filtered, and distributed in pipes throughout the city.

**Medico-Chirurgical Transactions.** Vol. LXXXI. London: Longmans, Green and Co. 1898.—Such a deservedly high reputation belongs to the work of this society, that it is hardly necessary to do more than to say that this present volume contains many papers of great interest. Perhaps their chief characteristic is that they all deal with subjects of direct practical importance in medicine and surgery.

**Transactions of the Medical Society of London.** Vol. XXI. London: Harrison and Sons. 1898.—The present volume contains some interesting and important papers, among which we would especially notice Cases of Operation on Pancreatic Cysts, by Mr. Alban Doran, Dr. H. G. Rolleston, Mr. G. R. Turner, and Dr. J. D. Malcolm; also a paper on "Rectal Surgery," illustrated with numerous drawings, by Mr. Thomas Bryant. The reports on the discussions at the Society's meetings are, as usual, full and instructive.

**Transactions of the Association of American Physicians.** Vol. XIII. Philadelphia: Printed for the Association. 1898.—Medical subjects of present interest are dealt with in these papers, which uniformly maintain a high level of excellence, and represent a large amount of original work and observation. Exigencies of space forbid our discussion of individual papers, and we must be content with commending the book to the careful perusal of our readers. Many of the contributions are very well illustrated.

**Transactions of the Michigan State Medical Society.** Vol. XXII. Grand Rapids: Published by the Society. 1898.—The volume is an account of the thirty-third annual meeting of the Society, held at Detroit, and consequently contains papers and discussions on most branches of "medicine"—papers differing in merit, of necessity, but many of which can be read with interest and profit. The printing has not been carefully corrected. "Program" is an Americanism we do not mind accepting, as we already have "diagram," but not so with "French capitol" (p. 24), "hair lip" (p. 13), and "preperation" (p. 192). An amusing *lapsus* occurs on p. 13, where it is said of Celsus (who was not an American, or we should have thought less of it) that "in wounds of the intestines, he performed gastrorrhaphy."

**Transactions of the South Carolina Medical Association.** Charleston: Lucas & Richardson Co. 1898.—In the opinion of the President the general appearance of the *Transactions* could be greatly improved. That is so, and the proof-reading also; we noticed "Opthalmology" (pp. 3 and 7), "Diptheria" (p. 25), and "parisites" (p. 187). The book should have a cloth cover; and if the sheets must be wired, it should not be done through their sides. Among the many good papers in this record of the forty-eighth meeting we were specially attracted to one on "Serum Diagnosis of Typhoid Fever," by Dr. Robert Wilson, jun., who reflects the general opinion when he says that the Widal test is one the usefulness of which "no physician who lays claim to scientific attainment can afford to ignore."

**The Transactions of the Edinburgh Obstetrical Society.** Vol. XXIII. Edinburgh: Oliver and Boyd. 1898.—The valedictory address of Dr. Alexander Ballantyne, contained in this volume, makes a pleasing reference to Spencer Wells and to the general work of the Society during the present year. It is perhaps not unnatural that some remarks should have been made on professional secrecy, as the case of Kitson *v.* Playfair and Wife had recently been before the public, and the retiring president's words may be read with profit. Among the papers is one by Dr. John Moir, a veteran practitioner of 90 years, on the induction of premature labour, another by Dr. J. W. Ballantyne on a vitelline placenta in the human subject, and a description of a sireniform fœtus. The other communications are of great value.

**Saint Thomas's Hospital Reports.** Vol. XXVI. London: J. & A. Churchill. 1898.—Besides a number of papers dealing chiefly with cases of interest that have occurred in the hospital, there are the usual full reports of the medical and surgical sides and of the various special departments. A full abstract of cases of special importance is also given. Dr. Payne has a very interesting contribution on some old physicians of St. Thomas's Hospital, with portraits of Dr. Wharton and Dr. Mead. A new department for physical exercises has been added to the hospital, and should prove a very excellent departure, which might well be copied by other hospitals.

**Tuberculosis.** Vol. I., No. 1. October, 1899. London: 20 Hanover Square.—This modest-looking new journal, issued by the "National Association for the Prevention of Consumption and other forms of Tuberculosis," is destined to perform good service in disseminating the proceedings of the association. Sir Hermann Weber's views on the production and prevention of tuberculosis would alone give the journal every title to a friendly welcome.

**Notes on Surgery for Nurses.** By Joseph Bell, M.D. Fifth Edition. Pp. 194. Edinburgh: Oliver and Boyd. 1899.—The fifth edition of this useful book has been thoroughly revised, and will prove a valuable text-book for the nurses' library.

**Case Paper.** Designed by Drs. J. K. Couch and E. Le C. Lancaster. Bristol: John Wright & Co.—These papers are of the usual type, but with the advantage that they have at the back outline figures for all parts of the body. They are sold separately, or bound up in books of 250, indexed and numbered. For those who like forms for case-taking this one should be most convenient and useful.

**Burdett's Hospitals and Charities.** London: The Scientific Press (Limited). 1899.—This hardy annual is, as usual, brimful of useful information; but as we have said on previous occasions, we attach far more importance to the statistical tables than the commentary upon them or the deductions drawn from them. Last year Sir Henry strongly advocated the payment of medical men for their services at hospitals: this year the matter is consigned to oblivion, and possibly wisely dropped; but it is replaced by the author's views on payments by patients, to which he has been converted. This is a feather in the cap of Mr. Sidney Holland, of the London Hospital, who has always supported this system; but we should not be surprised if this was next year replaced by some other plan for establishing our hospitals on a more sound financial basis. With regard to local matters, we find no reference to the Bristol Hospital Sunday Fund, which has been two years in existence on its present basis. We suggest to Sir Henry that he should apply for a properly-audited balance-sheet of any institution included in his annual, and that he should not be led away by the names of titled people as patrons.

**The International Directory of Booksellers and Bibliophile's Manual.** Rochdale: James Clegg. 1899.—This is a carefully-compiled work, containing in alphabetical order the names and addresses of booksellers, publishers, book-collectors, etc., all over the world. There is other information, such as articles on book-plates, copyright registry, sizes of books, etc. The need of such a book must be limited, but it will be found useful to librarians and lovers of books. The only relief from the "seriousness" of the work is the "Book-Lover's Lexicon," wherein a few newly-coined words will be found. The "book borrower who carries off your choicest treasures and resents any suggestion as to their return as the deadliest insult" may be known to many of us, but there must be few who know that such an one is correctly described as a "bibliopokomist."

---

